# Multifractal Properties of a Closed Contour: A Peek beyond the Shape Analysis

**DOI:** 10.1371/journal.pone.0115262

**Published:** 2014-12-26

**Authors:** Paulo Duarte-Neto, Borko Stošić, Tatijana Stošić, Rosangela Lessa, Milorad V. Milošević, H. Eugene Stanley

**Affiliations:** 1 Departamento de Estatística e Informática, Universidade Federal Rural de Pernambuco, Recife, Pernambuco, Brazil; 2 Departamento de Pesca e Aquicultura, Universidade Federal Rural de Pernambuco, Recife, Pernambuco, Brazil; 3 Departement Fysica, Universiteit Antwerpen, Antwerpen, Belgium; 4 Center for Polymer Studies and Department of Physics, Boston University, Boston, Massachusetts, United States of America; Fondazione Edmund Mach, Research and Innovation Centre, Italy

## Abstract

In recent decades multifractal analysis has been successfully applied to characterize the complex temporal and spatial organization of such diverse natural phenomena as heartbeat dynamics, the dendritic shape of neurons, retinal vessels, rock fractures, and intricately shaped volcanic ash particles. The characterization of multifractal properties of closed contours has remained elusive because applying traditional methods to their quasi-one-dimensional nature yields ambiguous answers. Here we show that multifractal analysis can reveal meaningful and sometimes unexpected information about natural structures with a perimeter well-defined by a closed contour. To this end, we demonstrate how to apply multifractal detrended fluctuation analysis, originally developed for the analysis of time series, to an arbitrary shape of a given study object. In particular, we show the application of the method to fish otoliths, calcareous concretions located in fish's inner ear. Frequently referred to as the fish's “black box", they contain a wealth of information about the fish's life history and thus have recently attracted increasing attention. As an illustrative example, we show that a multifractal approach can uncover unexpected relationships between otolith contours and size and age of fish at maturity.

## Introduction

The objects of classical shape analysis, such as Fourier analysis, wavelet analysis, curvature-based analysis, and geodesic curve analysis [1], [2], are composed of compact differentiable manifolds, smooth curves or surfaces that include their boundaries. In this view, natural contours consist of a superficial coating of texture or irregularity that is attached to a compact underlying structure. Hence, rough contours can be decomposed into smooth differentiable trends and rough additions [3]. In the case of fractal theory, roughness is considered as the main feature evaluated, since it captures the complexity of the shape in terms of the level of protrusions and cavities at different scales, rather than shape in the sense of morphometry. This characteristics is important because variations in the boundary of a natural structure during growth is a response to (i) external boundary conditions (surface interaction) and (ii) the internal mechanisms of the growth process. Therefore, the analyses of local and global fluctuations of the contour may provide useful information on both.

However, when we assume a contour shape is a monofractal, we get only a single scale exponent (fractal dimension), which cannot adequately describe contour complexity. Thus a generalized multifractal approach is needed [4]. If we consider the mass probability 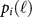 for regions of size (scale) 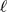, and the partition function 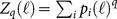 for 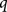
th generalized moments, we can describe the structure at different scales. Parameter 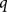 serves as a magnifying glass: for large positive 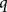 the partition function 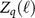 is dominated by those parts of the structure with the largest values of 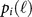, while for large negative 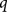, 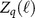 is dominated by the parts of the structure with the smallest (non-zero) values of 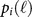 [5].

In recent decades multifractal analysis has been successfully applied to characterize the complex temporal and spatial organization of very diverse natural phenomena, including heartbeat dynamics [6], the dendritic shape of neurons [7], retinal vessels [8], rock fractures [9], and intricate shapes of volcanic ash particles [10]. Nevertheless, practical difficulties have thus far prevented the full use of multifractal analysis to describe closed contours. The traditional techniques have been demonstrated to be rather problematic because of the fact that boxes which contain a small (or zero) number of particles (or pixels) give an anomalously large contribution to the partition function, and consequently they do not yield reliable results for negative 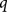 [7]. Another problem is that results turn out to be very sensitive to the choice of the box size range. Tél et al. [11] proposed an alternative method (the “Generalized Sand Box Method”) to solve the first problem, nevertheless the second issue still remains problematic. These methods also assume the contour is a geometrical fractal, and thus important fine fluctuations around the quasi-one-dimensional structure of the contour perimeter may be ignored.

**Figure 1:**
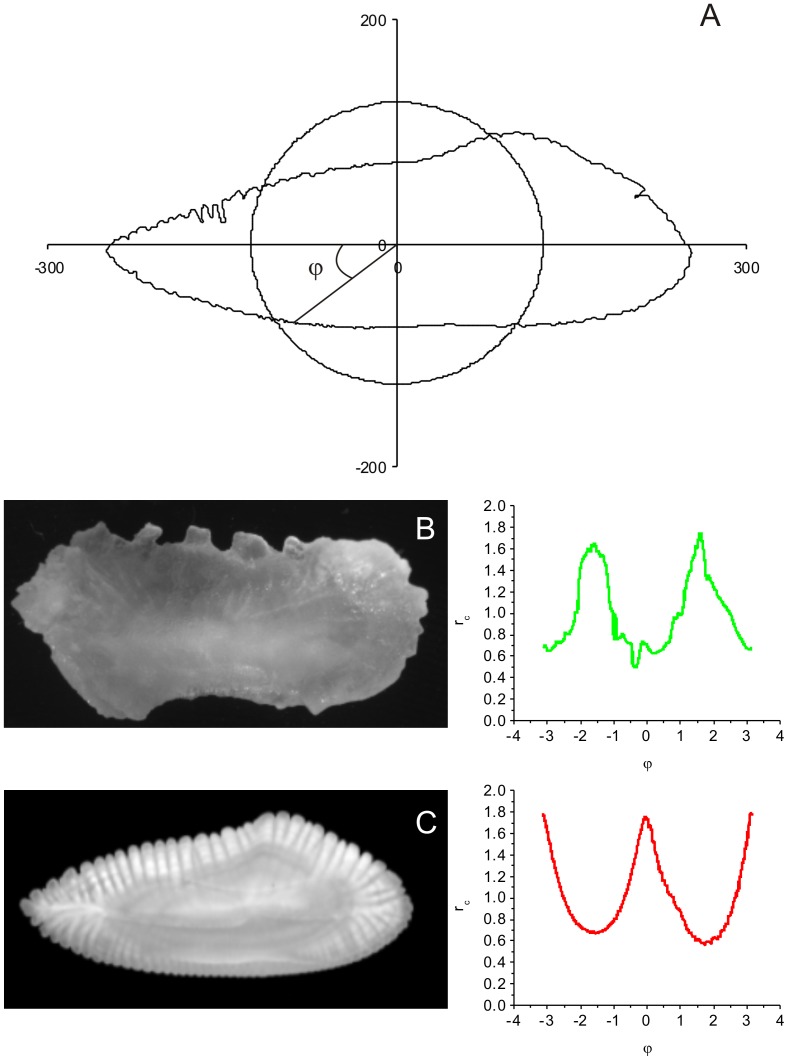
Periodic series of otolith contour fluctuations. (A) Schematic representation of the superposition of the standard circular shape on an otolith of *M. merlucius*. The otolith radius was used to define the periodic series of the otolith contour fluctuations, derived from the normalized radius 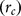 of the contour at the angle 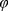 for (B) *M. curema* and (C) *M. merlucius*, obtained from the image catalog of the project AFORO.

To overcome these technical problems, we propose here a new technique to investigate whether fluctuations of the contour can reveal more information than its bare morphological appearance, combining Regular Fourier Analysis (RFA) and Multifractal-Detrended Fluctuation Analysis (MF-DFA). First, the contour is mapped onto a “time series” of distances from the central path, defined by harmonic term zero (Fig. 1A) as observed by a virtual observer traveling along this path at constant angular speed. The fluctuations from the central path of the contour perimeter are registered as a “time series”, and the MF-DFA is then implemented to quantify the “temporal” (sequential angular) correlations of this series.

To demonstrate the power of the proposed novel procedure, we apply it to sagittal otoliths of two fish species (Fig. 1B and C). Otoliths are calcified concretions found in a fish's inner ear and are associated with the functions of hearing, balance, and orientation [12]. They represent the “black-box” of teleost fishes, i.e., they function as an encrypted source of life history, demographic, and ecologic information [13], and are considered indispensable in fish stock evaluation and management practice [14]. Information is stored in the otolith during its biomineralization process, which starts at the otolith primordium [13] and continues with the precipitation of calcium carbonate regulated by the endogenous rhythm of calcium metabolism [15]. Over the fish's life, the rhythm of calcium aggregation changes, reflecting the growth pattern of the fish and such periodic events as photoperiod variation, spawning, and migration [16]. These changes are reflected in the formation of micro- and macro-structures around the primordium [17], the chemical composition, thickness, and periodicity of formation, which are correlated with historic events and with the age of the fish [16].

## Material and Methods

### Data series construction

The data series is constructed using the values of the radius of the contour 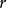 at the angle 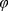, normalized by the zero-th harmonic (Fig. 1A), with 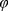 varying between 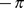 and 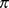. The normalized (dimensionless) contour radius 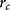 at point 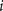 of the contour 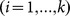 is defined as
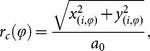
(1)where 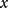 and 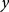 are the coordinates of the 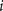 th contour pixel at the angle 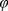, and 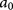 is the coefficient of zero-th degree term, defined as the mean of the 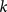 radii observed in the structure, 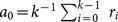 [18]. The zero-th degree term represents the contribution of a circle centered on the center of mass of the structure. Therefore 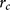 is less than one if the contour point lies inside the circle, and it is greater than unity if the point lies outside the circle.

There are two advantages of using the RFA: (1) the multifractal analysis becomes invariant to size, since the zero-th degree term is proportional to the size of the image; (2) complex morphological contour may present multiple values for a single angle due to protrusions and cavities. The last feature is in fact commonly considered a limitation for the use of RFA, but it is precisely the opposite in the current approach, because this effect induces noise into the data series, and the multifractal characteristics become more pronounced. That is, at the same angle, the structure could present sites with high, moderate and low probability of aggregation, that characterize the complexity of the analyzed structure.

The current mapping of the data may be considered as a time series of the values of the distance from the actual contour to the basic regular shape (defined by the zero-th harmonic), as seen by an observer traveling along the regular shape at constant angular speed. Using this time series, the multifractal analysis is carried out based on the MF-DFA method proposed by Kantelhardt et al. [19] to analyze multifractal properties of non-linear temporal series.

### Multifractal Detrended Fluctuation Analysis (MF-DFA)

Let 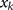 a periodic series of 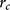 values between 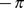 and 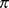, of length 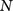, corresponding to the number of pixels that form the contour, having mean 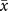.

(i) First an integrated series 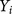 is calculated as
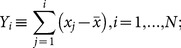
(2)


(ii) The integrated series 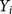 is divided into 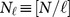 non-overlapping segments of equal length 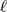, where symbol 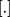 stands for integer part.

(iii) For all of the 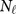 segments the fluctuation function 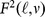 is calculated as
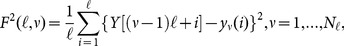
(3)where 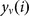 is the fitting polynomial in segment 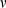, representing the local trend.

(iv) The fluctuation function of 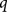 th degree for segment size 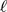 is given by
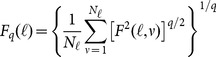
(4)


In theory 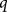 can assume values between 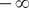 and 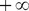, but in practical applications it is truncated at some large positive and negative values. In this work, the multifractal properties were analyzed in the interval of 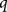 between −10 and 10, with steps of 1.0. The minimum segment size used was 15 data points (corresponding to otolith contour pixels) and the maximum was adopted as one fourth of the total number of points of the series.

**Figure 2:**
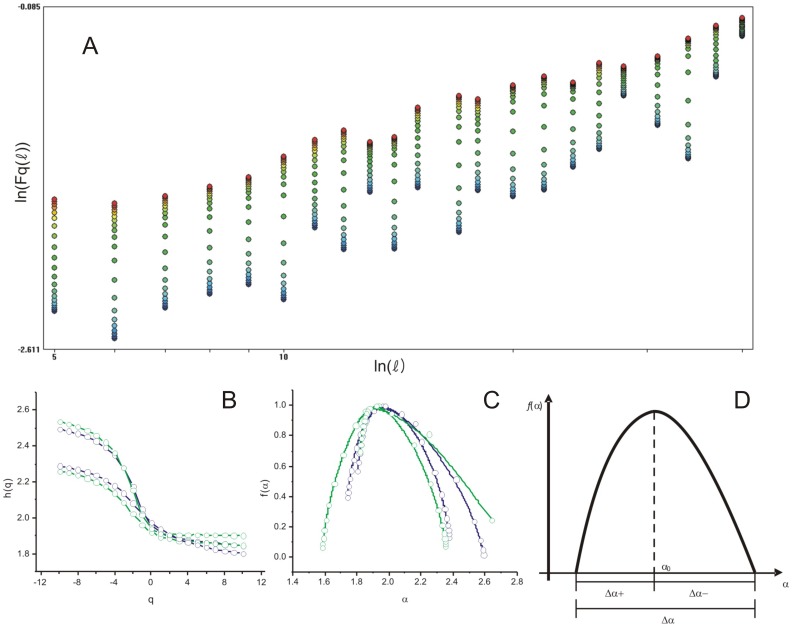
Multifractal plots derived from the MF-DFA of otolith periodic series. (A) The linear regression between 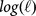 and 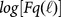. (B) The 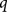 th moment versus the generalized exponent 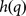 determined as the slope of (A). (C) The singularity spectra 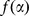 derived from the fluctuation contour of two *M. merluccius* (blue) and two *M. curema* (green) otoliths. (D) Schematic representation of the multifractal parameters extracted from the singularity spectrum.

(v) The function 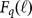 represents the partition function for this multifractal analysis and follows a power law
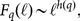
(5)where the generalized exponent 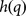 is the slope of the linear regression between 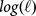 and 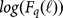 (see Fig. 2A). For a monofractal process 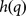 is constant (independent of 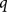), and for a multifractal process 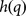 is a decreasing function of 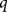 (Fig. 2B).

As is common in the literature using the MF-DFA approach, besides the functional form 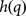, the multifractal properties of contours are also investigated based on the so called singularity spectrum 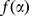, achieved through the Legendre transform
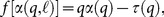
(6)where
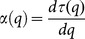
(7)and 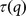 is the mass correlation exponent of the 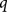 th moment, defined as 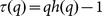. The singularity spectrum provides a mathematically precise and naturally intuitive description of the multifractal measure in terms of interwoven sets with singularity strength 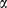, whose Hausdorff dimension is 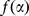 (see Ref. [5] for more details). In the case of a monofractal structure, the singularity spectrum produces a single point in the 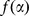 plane, whereas multifractal objects yield a single humped function (Fig. 2C).

A set of parameters can be extracted from the multifractal spectra (*cf*. Fig. 2D) for characterizing contour complexity, each with a clear intuitive interpretation. For example, 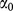, the position of the maximum, is low if the signal is uncorrelated and the underlying process “loses fine structure" (i.e. the dominant fractal structure has more energy at larger fluctuation, since fine fluctuations become less frequent). The width 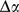 measures the range of the fractal exponents in the signal, i.e., the wider the range, the more multifractal are the contour fluctuations. 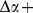 and 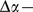 measure the dominance of low and high fractal exponents, respectively: a larger 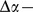 indicates strong weight of high fractal exponents, corresponding to a fine structure in the contour, while a larger 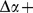 indicates higher fluctuations in the series, i.e., there are large structures in the contour. Finally, the ratio between 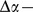 and 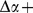, labeled 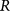, represents the relative dominance of these two parameters. This five-dimensional parameter space may therefore be used to characterize the complexity of the contour.

### Image Sample

The sample was composed of 65 high-resolution otolith images of *Mugil curema* (Fig. 1B) from the north region of Pernambuco (Brazil), and 32 of *Merluccius merluccius* (Fig. 1C) from Port de La Selva (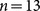) and Galcia (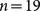). All *M. curema* were collected from landings of the artisanal fleet operating in the state of Pernambuco (northeastern Brazil) from November 2003 to January 2006. Specimens of different sizes were purchased directly from fishermen and taken to laboratory to posterior measurement of the total length and otolith extraction. After cleaned and dried, otolith images were captured using a charge-coupled device camera mounted on a microscope and processed using an image-analysis system developed for calcified structures (TNPC: Visilog software platform, NOESIS, France). *M. merluccius* otolith images were obtained from the open online catalogue of otolith images of the project Anàlisi de FORmes d′Otòlits - AFORO [20].

### Ethics Statement

The artisanal fisheries of *M. curema* represent a legal activity in the state of Pernambuco (Brazil), since this species is not classified as endangered or protected. All non-living specimens was purchased directly from fishermen, avoiding the use of any method of sacrifice by the authors. Consequently, no specific permission was required from animal ethics committee to conduct the present work, including fish sampling and posterior otolith extraction.

## Results and Discussion

**Figure 3:**
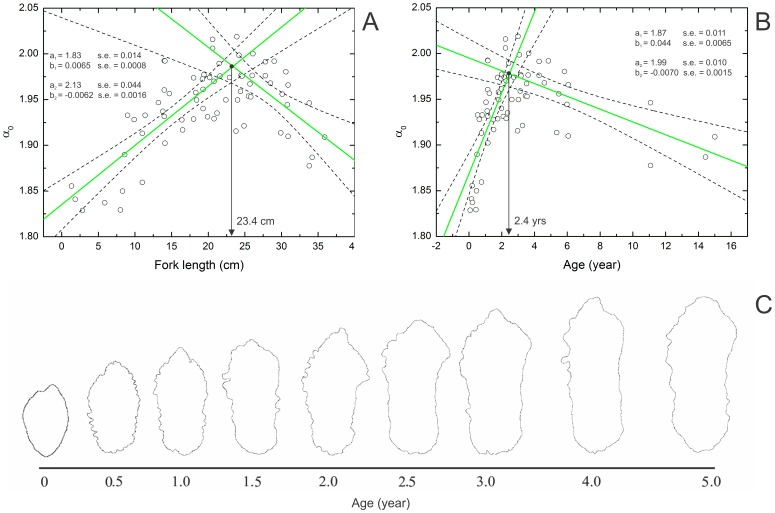
Plot of 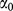 parameter against length and age of *M. curema*. (A) Length and (B) age variation of individuals from the north region of Pernambuco (Brazil). Green lines represent the linear fit to the two subsets of the data (1-left and 2-rigth to the maximal 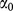), with respective parameters of the fit (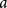 - intercept and 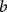 - slope) and 95% confidence bands (dashed lines). The black arrow indicates the interception between the two lines. (C) Schematic representation of the roughness variation of the sagittal otolith contour of *M. curema* as a function of age.

The multifractal analysis of otolith contours in two species reveals clear multifractal behavior. The generalized exponent 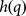 presents a monotonic decay with 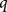 (Fig. 2B), and the singularity spectrum has a humped shape (Fig. 2C). An observation of individual length and age provides solid proof that the multifractal properties of an otolith contour reflects life history events. For *M. curema*, the 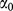 distribution shows a peak at 23.9 cm fork length (Fig. 3A) and age of 3 years (Fig. 3B). We fitted two lines (using ordinary least square method) to both length and age data (Fig. 3). The first line was fitted to individuals with 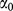 left to the observed maximum, and the second one was fitted to individuals with 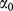 right to the maximum. The intercept of these two lines was found at 23.4 cm (Fig. 3A) and 2.4 years (Fig. 3B). Thereby obtained peaks closely correspond to the expected length (23.3 cm) and age (2.8 years old) at first sexual maturity for both sexes, as documented previously by Santana et al. [21] using gonads. Note that here we observed no difference between the growth of males and females.

On the other hand, plotting 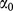 versus length/age for *M. merluccius* reveals two different sub-patterns. We attribute this behavior to the fact that this species has different growth rates between sexes, with males growing more quickly than females [22], [23] and reaching maturity at different sizes and ages. From the biological point of view, the maximum 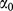 observed for each species, or sex/population within one species, seems to be a function of the growth rate, i.e. faster growth implicates a higher maximal 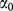 (rougher otoliths).

**Figure 4:**
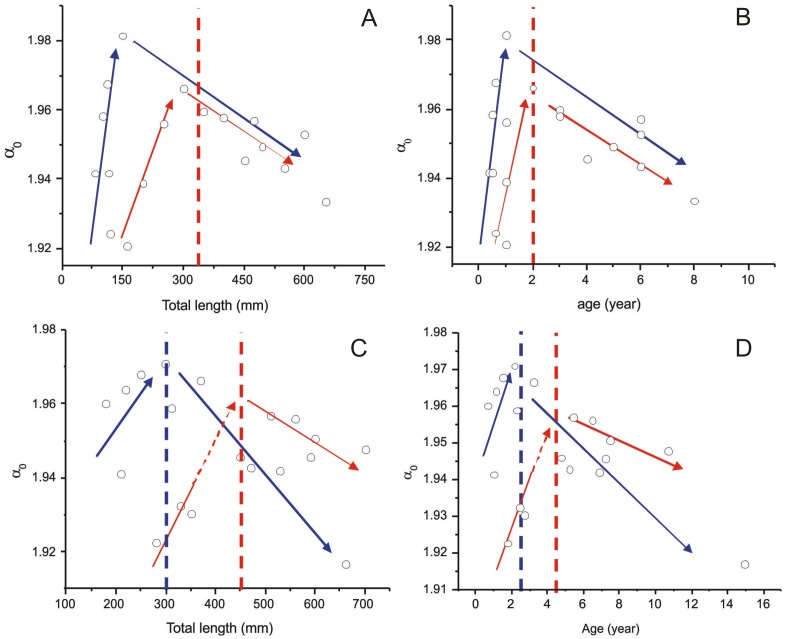
Plot of 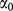 parameter against length and age of *M. merluccius*. (A and B) Individuals from Port de La Selva (Mediterranean population) and (C and D) individuals from Galícia (Northeastern Atlantic population). Blue and red arrows represent the variation pattern of the 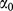 for males and females, respectively. Vertical red and blue lines indicate the length and age of first maturity for females and males, respectively, estimated earlier by different authors.

For the Mediterranean population 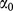 values show two peaks around 15.0 cm and 30.0 cm (total length) (Fig. 4A) and the corresponding peaks in age were at one and two years respectively (Fig. 4B). A recent study of the reproductive pattern of *M. merluccius* from the Mediterranean Sea estimated the length at first maturity of females to be approximately 35.0 cm [24], which is very close to the second peak in our analysis. Using the growth parameters determined by Mellon-Duval et al. [22] for females, the corresponding age at first maturity is two years, precisely the age with the highest 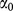 value in our analysis. The two maximal 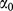 values for the Atlantic population were found at 30.0 and 45.0 cm total length (Fig. 4C), which match closely the lengths of first maturity estimated by Piñeiro and Saínza [23] for males (32.8 cm) and females (45.0 cm) respectively. The linear fitting procedure was not carried out here because of the small available number of experimental observations.

Factors that influence somatic growth, such as temperature, salinity (environment), and hormone levels during development, growth and reproduction of fishes (physiology), also affect otolith growth [25]. In function of these factors, fishes and other organisms change the metabolism rate to assimilate and utilize energy for maintenance, growth, development and reproduction throughout their life cycle, all of which reflect on the otolith growth [26]. It is likely that there is limited precision in the *M. merluccius* analysis due to the reduced number of individuals in the available dataset. Nevertheless, it is clear that changes in the otolith contour during the fish's life caused by alteration of the metabolic rate between reproduction and somatic growth is captured by the multifractal analysis. For these species, the changes are reflected in the 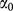 parameter, meaning that the sagittal otolith roughness level follows the fish growth, as illustrated in Fig. 3C, while the general shape is kept unchanged.

For all species, the rhythm of calcium carbonate precipitation, controlled by the fish metabolism, increased the roughness of the otolith contour until the maturity of the fish, due to a faster growth during this phase. Then, from this point onwards, the growth of the fish and the otolith become slower, and, consequently, the otolith becomes smoother, despite the more complex shape (larger fluctuations). Furthermore, different growth rates for sexes due to the physiological factors are also indicated by the 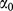 plots (Fig. 4), as well as different observed growth between different populations.

Length at first maturity (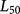) represents a reference point for fish biology and its estimation is useful for fish stock management. For instance, the minor size of capture can be established based on 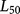, aiming to avoid the over-exploitation of a fish stock. Note that the determination of first sexual maturity of fish has to date been possible only through costly and cumbersome experimental techniques [21]. Different methods have been proposed to estimate 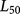 [27], [28]. The differences among these methods reduce to just the used statistical approach, however, the common biological structure for all of them was the gonad. In most of the techniques, individuals are identified as reproductive or non reproductive, through visual and subjective descriptions of macroscopic aspects of ovaries and testicles at different maturation stages, or based on the Gonadosomatic Index [28]. However, gonads are sometime unavailable for commercial species, since fishes are eviscerated before landing. The method presented here appears to be a useful alternative procedure, filling the information gap that could exist for any fish populations based only on previously available data. Therefore, the multifractal analysis of otolith contours should prove to be an important tool in fish stock evaluation and management.

Fractal dimension of otolith contour was previously estimated by Piera et al. [29] and Duarte-Neto et al. [30] for the purpose of otolith classification, using box-counting method. Contradictorily, fractal dimension was found unable to classify otoliths of *M. merluccius* of different ages in the first work, whereas it was a powerful descriptor in discriminating otoliths of two stocks of *Corypahena hippurus* in the second. This single exponent obtained in both works represents global properties of the contour and says nothing about the local properties. *C. hippurus* otolith presents in general a complex shape and low level of roughness. On the other hand, *M. merluccius* otolith presents a simple ellipsoidal shape, with few large fluctuations, and is very rough. Analyzing otolith contours on the basis of multifractal method described here allows the description of the complexity of their shapes in more detail, from fine to large scales, based on the distribution of the multifractal morphological exponents.

Box-counting method may also be employed in the multifractal approach, as well as the Sanding box method. Although the multifractal versions of these methods are well established in the scientific community, they do not seem appropriate for analyzing quasi-one-dimensional structures, with complexity far from filling two dimensional space, but more complex than a line. For instance, the contour that displays such characteristic is the one of the *C. hippurus* otolith [30], with maximum fractal dimension of 1.248. On the other hand, the methods commonly used for multifractal analysis of time series do not have these types of problems [19], [31], [32]. Among them, MF-DFA yields reliable results both for large negative q and for shorter signals [32], besides having lesser requirements for computational power [19]. This method was demonstrated to be rather satisfactory for analysis of otolith contour fluctuations in the current work, and no problems have been experienced in the implementation. Still, image resolution could be a limitation for it use, since a higher resolution should exhibit more particularities of the images. To avoid possible complications due to such an effect, all the images analyzed here were taken at the same resolution.

Since our current approach is essentially general, it could be applied to contour studies of other natural structures. For example, the multifractality of mineral particles has been assessed so far only in terms of their spatial arrangement in soil [33], [34], and such a shape characterization would also be important in the classification of different types of sediment [35] and ash particles [10], as well as biological entities such as corals and cells. How the multifractal properties of contour fluctuations behave during particle formation, and how they relate to various growth and parallel processes, is still not well understood, and the current “traveling observer” MF-DFA approach may prove useful for the elucidation of such phenomena.
